# S-adenosylmethionine upregulates the angiotensin receptor-binding protein ATRAP via the methylation of HuR in NAFLD

**DOI:** 10.1038/s41419-021-03591-1

**Published:** 2021-03-22

**Authors:** Tao Guo, Zhe Dai, Ke You, Shyue-Fang Battaglia-Hsu, Juan Feng, Fengliang Wang, Bao Li, Jian Yang, Zhen Li

**Affiliations:** 1 Department of Pathophysiology, School of Basic Medical Sciences, Weifang Medical University, 261053 Weifang, China; 2 Department of Endocrinology, Zhongnan Hospital of Wuhan University, 430071 Wuhan, China; 3 Department of Hepatobiliary Surgery, The Second Affiliated Hospital of Chongqing Medical University, 400010 Chongqing, China; 4 Nutrition, Genetics, and Environmental Risk Exposure, Faculty of Medicine of Nancy, University of Lorraine and University Regional Hospital, 54000 Vandoeuvre-lès-Nancy, France; 5 School of Health Sciences, Wuhan University, 430072 Wuhan, China; 6 Department of Breast Surgery, The Affiliated Obstetrics and Gynaecology Hospital of Nanjing Medical University, Nanjing Maternity and Child Health Care Hospital, 210004 Nanjing, China; 7 Department of Urology, Weifang People’s Hospital, 261000 Weifang, Shandong China; 8 School of Nursing, Huanggang Polytechnic College, 438002 Huanggang, China; 9 Department of Hepatobiliary and Pancreatic Surgery, Zhongnan Hospital of Wuhan University, 430071 Wuhan, China

**Keywords:** Lipidomics, Metabolomics

## Abstract

Nonalcoholic fatty liver disease (NAFLD) has emerged globally and is associated with inflammatory signaling. The underlying mechanisms remain poorly delineated, although NAFLD has attracted considerable attention and been extensively investigated. Recent publications have determined that angiotensin II (Ang II) plays an important role in stimulating NAFLD progression by causing lipid metabolism disorder and insulin resistance through its main receptor, Ang II type 1 receptor (AT1R). Herein, we explored the effect of supplementary *S*-adenosylmethionine (SAM), which is the main biological methyl donor in mammalian cells, in regulating AT1R-associated protein (ATRAP), which is the negative regulator of AT1R. We found that SAM was depleted in NAFLD and that SAM supplementation ameliorated steatosis. In addition, in both high-fat diet-fed C57BL/6 rats and L02 cells treated with oleic acid (OA), ATRAP expression was downregulated at lower SAM concentrations. Mechanistically, we found that the subcellular localization of human antigen R (HuR) was determined by the SAM concentration due to protein methylation modification. Moreover, HuR was demonstrated to directly bind ATRAP mRNA and control its nucleocytoplasmic shuttling. Thus, SAM was suggested to upregulate ATRAP protein expression by maintaining the export of its mRNA from the nucleus. Taken together, our findings suggest that SAM can positively regulate ATRAP in NAFLD and may have various potential benefits for the treatment of NAFLD.

## Introduction

The prevalence of nonalcoholic fatty liver disease (NAFLD), which is specified through a broad spectrum of liver damage ranging from simple steatosis to advanced fibrosis, steatohepatitis, and cirrhosis in the absence of excessive alcohol consumption^1^, is unfortunately increased in patients with obesity and other metabolic syndromes, and this disease is expected to be the leading cause of liver failure in the future^2^. The progression of NAFLD includes severe stages, such as nonalcoholic steatohepatitis (NASH), which can lead to more severe liver diseases, such as cirrhosis and hepatocellular carcinoma (HCC)^3^. More importantly, NAFLD has a high prevalence, and a significant portion of cases are pediatric cases^4,5^. Current predictions suggest that the number of obese patients with NAFLD will increase in the next decade, which indicates that NAFLD will place a heavy burden on public health systems^6^. Unfortunately, the molecular mechanisms driving hepatic steatosis and the pathological processes that cause NAFLD progression are still poorly understood^7^.

According to basic medical studies, many factors are involved in the NAFLD progression. For instance, accumulating evidence has proposed that the renin–angiotensin system (RAS) is involved in hepatic fibrogenesis and inflammation^8–10^. In the RAS, angiotensin II (Ang II), the leading biologically active product of the RAS, mostly targets two types of cell surface receptors, type 1 receptor (AT1R) and type 2 receptor (AT2R), with AT1R being the main receptor subtype. Ang II/AT1R was determined to promote NAFLD, and previous studies have illustrated that AT1R blockade can ameliorate fatty liver and that AT1R blockers can potentially be used for NAFLD therapy^11,12^. Moreover, AT1R-associated protein (ATRAP, also known as AGTRAP), as a molecule that directly interacts with the carboxyl-terminal domain of AT1R, functions as a negative modulator of AT1R activation. ATRAP can inhibit the biological effects of Ang II though AT1R binding^13–16^. Additionally, ATRAP was shown to potentially prevent tissue metabolic abnormalities, including lipid deposition and hepatic fibrosis^17,18^. Thus, ATRAP was deemed a promising negative regulator of NAFLD.

In contrast, the liver performs a fundamental task in the metabolism of *S*-adenosylmethionine (SAM), which is the main biological methyl donor produced in all mammalian cells^19,20^. As a methyl supplement, SAM participates in multiple biological processes and alters chemical modifications of the genome and proteins^21^. SAM has been demonstrated to provide clinical benefits in chronic liver disease^22^. More importantly, SAM induces the methylation of phospholipids, which performs a crucial task in the metabolisms of lipids^23^. These findings suggest that a chronically changed hepatic SAM level could be a trigger that promotes NAFLD^20,24^. In addition, animal models and clinical observations have also implied that SAM exerts protective effects against NAFLD^25,26^. However, to date, the mechanisms by which SAM regulates NAFLD remain largely unknown. The interaction between SAM and ATRAP in NAFLD is also of interest. In this research, we aimed to discover specifically how SAM regulates ATRAP in NAFLD through a deep investigation of the molecular mechanisms to enrich our understanding of NAFLD.

## Methods

### Ethical application and clinical data collection

The relevant protocols were confirmed through the Human Subjects Committee of Zhongnan Hospital of Wuhan University and Second Affiliated Hospital of Chongqing Medical University conforming to the Declaration of Helsinki. Letter of informed consent was acquired from all patients if their samples were utilized. For clinical data collection, we recorded general information, including name, age, and sex, in the outpatient phase, and preoperative parametric hospitalization data, including liver function and triglyceride (TG) and total cholesterol (TC) levels, were also collected for further analysis.

### Tissue samples

In this study, we included 19 NAFLD liver tissue samples and 24 normal liver tissue samples from 43 patients. The specimens were obtained from patients with hepatic hemangioma who previously endured surgical resection, and we only collected the adjacent tissues from hemangioma lesions. The patients included met the following criteria: (1) ≥18 years old; (2) no previous SAM therapy; (3) no tumors except hepatic hemangioma; (4) no previous surgical history; (5) no history of alcohol consumption; and (6) no history of hepatitis virus infection. For the 24 patients without NAFLD, hepatic hemangioma was the only diagnosis, and they were confirmed to have no metabolic diseases. The NAFLD patients had a clear presence of lipid deposition with or without hepatitis, as determined by postoperative pathologic diagnosis. All related adjacent tissues from the hemangioma samples were intraoperatively resected and immediately preserved in liquid nitrogen, 4% polyformaldehyde, or RNA protectant solution.

### Cell culture

The normal hepatic cell line L02 was obtained from the Cell Bank of Type Culture Collection (Chinese Academy of Sciences, Shanghai, China). The identification and viability of cell lines were determined by a third-party biology service (GeneCreate Biological Engineering Co., Ltd). L02 cells were cultivated in the minimum essential milieu (Gibco, Carlsbad, CA, USA) supplied with 10% serum of fetal bovine (Gibco), as recommended, at 37 °C in an incubator with 5% CO_2_.

### Cell treatment

For the in vitro NAFLD model, L02 cells were starved in serum-free medium overnight. Then 1 mM oleic acid (OA) (Sigma-Aldrich) and bovine serum albumin (BSA) (Sigma-Aldrich) were added to the L02 cells. The control group was cultured in medium consisting only of BSA. L02 cells in the SAM treatment group were incubated with SAM (Sigma-Aldrich, A2408; 120 μM) mixed with OA solution. And the L02 cells were treated with SAM/OA mixture plus adenosine-2′,3′-dialdehyde (AdoX; Sigma-Aldrich; 20 μM) in the methylation inhibition group.

### Small interfering RNA (siRNA) transfection

To knock down human antigen R (HuR), two different siRNAs targeting HuR were generated and tested by GeneCopoeia (GeneCopoeia, Inc.). Transfection of the cells was carried out by using 20 nmol/l siRNA and utilizing Lipofectamine RNAiMAX reagent (Invitrogen) following the producer’s protocol.

### Quantitative reverse transcription PCR (RT-PCR)

Total RNA was extracted from cells and tissues utilizing the reagent of TRIzol (Invitrogen, Carlsbad, CA, USA) conforming to the protocol of the manufacturer. RNA samples with a 260/280 nm ratio >1.8 were considered quality RNA specimens. A total volume of 20 µl was used for RT reactions, and these reactions were carried out utilizing a kit of PrimeScript RT reagent (Takara) conforming to the protocol of the manufacturer. For quantitative PCR (qPCR), 2 µl of diluted RT product was blended with 10 µl of 2× SYBR Master Mix (Toyobo), 2 µl primers (10 µM) (Supplementary Table S1), and 6 µl of nuclease-free water in a final volume of 20 µl conforming to the instructions of the manufacturer. Further, amplification was accomplished with the system of iQ5 qPCR (Bio-Rad) with annealing at 60 °C for 30 s and denaturation at 95 °C for 30 s for 40 cycles. qPCR was executed in triplicate, containing nontemplate controls. β-Actin was utilized for normalization of expression, and 2^−ΔΔCT^ values were normalized to the β-actin levels.

### Western blot analysis

Tissues and cell samples were cryogenically shaking for 30 min in lysis solution with cOmplete Protease Inhibitor Cocktail (Roche, Boulogne-Billancourt, France). Moreover, nucleoproteins were extracted with NE-PER™ Nuclear and Cytoplasmic Extraction Reagents (Thermo Scientific) conforming to the protocol of the manufacturer. All extracted protein samples were thermally denatured and stored at −20 °C until use. For anti-methyl HuR antibody production, we used previously published methods. Briefly, antibodies against methyl HuR were produced against the dodecapeptide HHQAQ(DMA)FRFSPG (where DMA represents asymmetric dimethylarginine) with an extra amino-terminal cysteine (United Peptide, USA). Then, 2 mg of the coupled peptide was blended with 200 μg of an auxiliary peptide (IMMACCEL-R, Aptum, UK) and imperfect Freund’s auxiliary and injected into rabbits. Immune sera were generated for intervals of 2 weeks and tested on immunoblots. Next, on immobilized peptides, the antibodies were then affinity purified. For western blotting, protein specimens were divided by 10 or 15% sodium dodecyl sulfate–polyacrylamide gel electrophoresis (PAGE) and membranes of polyvinylidene fluoride (Millipore, USA) were used for electronic transferal. The incubation of the membranes with primary antibodies was accomplished (Supplementary Table S2) during the night hours at 4 °C. Subsequently, the membranes were rinsed and incubated with secondary antibodies (1:2000 for all, Proteintech) for 1 h. Normalization of each band was done regarding its corresponding β-actin band, and for nucleoproteins, H3 was used for normalization. Band Scan (Glyko Inc.) version 5.0 was utilized to quantitatively assess each band for normalization.

### Histological staining

Tissue samples were inserted in paraffin and stained with hematoxylin and eosin (HE). All tissue samples were separated to create 4-mm-thick slices, and the slices were stained with HE for 3 min and 5 s after dewaxing. Intraoperative fresh tissues were frozen for Oil Red O staining to observe lipid droplet accumulation. L02 cells were adjusted in 4% paraformaldehyde for 10 min and then stained with Oil Red O for 30 min. The cells were repeatedly stained for 2–3 min with hematoxylin and differentiated in 1% hydrochloric acid alcohol. For immunohistochemical staining, the slices were incubated in boiling 0.01 mol/l citric acid buffer for 15 min for antigen repair before being incubated with primary antibodies (Supplementary Table S2). After incubation with secondary antibody, DAB solution (Dako Denmark A/S) and hematoxylin were used for staining before sealing.

### Fluorescence in situ hybridization (FISH)

RNA FISH was used to observe relative subcellular localization and was carried out based on the protocol of Ribo FISH Immobilized Kit (RN: 10910; RiboBio). Briefly, after prehybridization buffer treatment, the ATRAP probe (RiboBio) or lipid probe (Invitrogen) mixture was diluted in hybridization buffer after removing the prehybridization buffer and was incubated overnight at 37 °C. The DNA was stained with 4,6-diamidino-2-phenylindole (DAPI) for 10 min prior to sealing. Molecular abundance and subcellular localization were determined under the same optical conditions with a Double Disc Laser Confocal Imaging System (Olympus).

### Immunofluorescence

Frozen slices of liver tissues and immobilized slices of L02 cells fixed with polyformaldehyde were incubated with a primary antibody against HuR (Supplementary Table S2) during the night hours at 4 °C with moderate agitation. For nucleus detection, the nuclear fluorescent dye DAPI (Sigma, Lyon, France) was used. Finally, the slides were rinsed four times in phosphate-buffered saline and mounted utilizing a minimal volume of milieu (DAKO, Carpinteria, USA).

### Ultra-performance liquid chromatography (UPLC)

SAM standards (Sigma-Aldrich) were utilized to create the standard curves. Standard solutions with concentrations of 20, 50, 100, 200, 500, 1000, and 2000 ng/ml SAM were contained in each batch of specimens. The UPLC-LC/mass spectrometry measurements were carried out utilizing a system of ACQUITY UPLC (Waters, Milford), which was conjugated with an API4000 tandem mass spectrometer (AB Sciex). The specimens were divided by a Waters ACQUITY UPLC1® BEH Amide (2.1 mm × 100 mm, 1.7-mm particle size) column. The temperature of the column was 40 °C, and the injection volume was 5 μl. The retention time was 1.64 ± 0.5 min. The experiment was replicated 3 times for each specimen, and the mean value was computed.

### RNA pull down

RNA pull down was carried out with a kit of Pierce Magnetic RNA-Protein Pull-Down (Thermo Scientific, USA). Briefly, biotin-labeled ATRAP mRNA and negative control RNAs were incubated with L02 cell lysates and streptavidin magnetic beads. The beads were washed, and the proteins were eluted and resolved by PAGE. Then, silver staining was conducted. Gel fragments were excised and analyzed by western blotting.

### RNA immunoprecipitation (RIP) measurement

RIP was accomplished by utilizing the kit of RNA-Protein Immunoprecipitation (Magna RIP™) conforming to the protocol of the manufacturer. Briefly, L02 cells at 80–90% confluency were scraped and lysed in 15-cm dishes of culture and then incubation on ice was done for 5 min. After washing and magnetic suspension, the HuR antibody was used for incubation. The assessment of RNA quality and RT were fulfilled utilizing a Nanodrop^TM^ spectrophotometer (Thermo Scientific) as well as a kit of EZ-Magna RIP (Magna). At last, qPCR was employed to assess HuR-bound RNA, which was normalized to the input and compared to IgG-bound RNA. After qPCR, agarose gel electrophoresis was executed to visualize the fragments of cDNA.

### Animal experiments

We constructed a NAFLD model by utilizing 8-week-old male C57BL/6 rats in vivo. Twenty-one rats were kept in a humidity- and temperature-controlled room (23 ± 1°C and relative humidity of 60 ± 5%) on a 12-h light/dark cycle and were randomized into 3 groups (7 in each group): the control group, high-fat diet (HF) group, and HF plus SAM group. The experiments lasted for 8 weeks for all the 3 groups. Rats in the control group were given a normal balanced diet (4.15% crude fat, WanQianJiaXing Biotechnology). Nonalcoholic fatty liver was induced in the HF group by feeding the animals a high-fat diet (60% fat, OpenSource Diets™). SAM (Nature Made) was purchased in tablet form, crushed into powder, and dissolved in tap water. The rats in the HF plus SAM group were given SAM (200 mg/kg) once daily by gavage in addition to the abovementioned high-fat diet. An equivalent volume of saline was given by gavage to the rats in the control and HF groups. After 8 weeks, all 21 rats were sacrificed, and their liver samples were resected for subsequent utilization.

### Statistical analysis

The dichotomous variables were measured through Fisher’s exact test or χ^2^ test. Student’s *t* test was done to compare the continuous variables between groups. Statistical differences between groups were studied using SPSS 22.0 computer program (IBM, Chicago, IL, USA), and plots were created utilizing GraphPad Prism 6.0 (GraphPad computer program, USA).

## Results

### The expression levels of SAM and ATRAP were altered in NAFLD

**Fig. 1:**
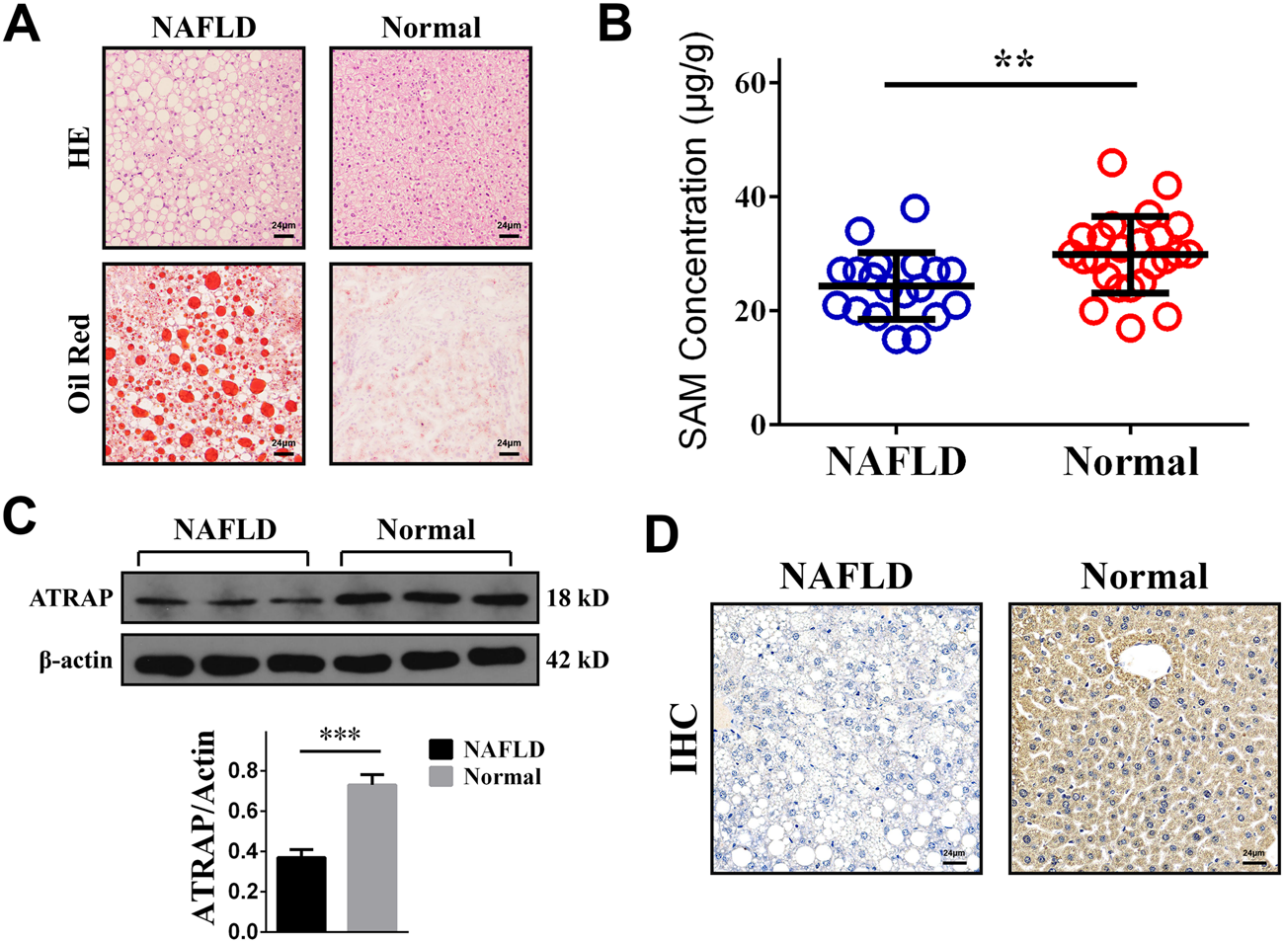
SAM and ATRAP expression was downregulated in NAFLD patients. **A** Images of clinical NAFLD and normal liver tissues stained with HE and Oil Red O (bar = 24 μm). **B** SAM concentrations in NAFLD and normal liver tissues, as determined by UPLC. **C** ATRAP protein levels in NAFLD and normal liver tissues, as determined by western blotting and **D** immunohistochemistry (bar = 24 μm). The error bars are the SDs (*n* = 3). **P < 0.01, ***P < 0.001.

We collected 19 NAFLD liver samples and 24 normal liver tissues from 43 patients who underwent hepatic hemangioma resection. We noticed no difference between the NAFLD and normal groups regarding preoperative characteristics except for body mass index and TG and TC levels (Table 1). The pathological features were observed by microscopic staining. Obvious lipid deposition was detected in NAFLD samples by HE and Oil Red O staining (Fig. 1A). Next, we investigated the SAM concentrations in these liver tissues and found that SAM was significantly depleted in NAFLD tissues compared to normal liver samples (Fig. 1B). We then measured the ATRAP protein levels in NAFLD samples by western blotting and immunohistochemistry. The results showed that, compared with that in normal tissues, ATRAP protein expression in NAFLD tissues was significantly downregulated (Fig. 1C, D). Thus, we found that both SAM and ATRAP levels were significantly lower in the liver tissues of patients with NAFLD than in those in normal liver tissues.

**Table 1:** The preoperative characteristics of the included patients.

	NAFLD (*N* = 19)	Normal (*N* = 24)	*P* value
Age (years)	50.58 ± 1.66	46.75 ± 2.04	0.170
Gender			
Male	9	10	0.764
Female	10	14	
BMI	27.38 ± 0.33	22.18 ± 0.33	<0.001*
Max tumor size (cm)	8.44 ± 0.86	7.06 ± 0.50	0.151
Smoke			
Yes	7	7	0.745
No	12	17	
ALT (normal)			
Yes	17	24	0.189
No	2	0	
AST (normal)			
Yes	17	24	0.189
No	2	0	
TG	2.44 ± 0.38	1.05 ± 0.06	<0.001*
TC	5.23 ± 0.50	4.15 ± 0.14	0.030*

Bold values represent statistical differences.

### SAM could inhibit downregulation of ATRAP expression in NAFLD

**Fig. 2:**
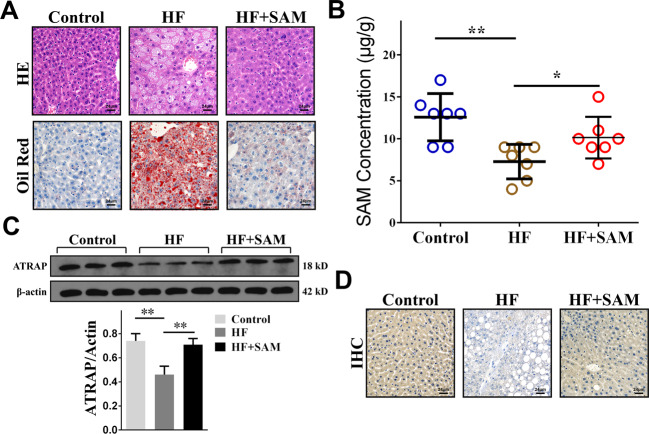
SAM could inhibit the downregulation of ATRAP expression in NAFLD. **A** Images of liver tissues of rats from the control, HF, and HF plus SAM groups stained with HE and Oil Red O (bar = 24 μm). **B** SAM concentrations in the liver tissues of rats in the control, HF, and HF plus SAM groups, as determined by UPLC. **C** ATRAP protein levels in the liver tissues of rats from different groups, as determined by western blotting and **D** immunohistochemistry (bar = 24 μm). The error bars are the SDs (*n* = 3). *P < 0.05, **P < 0.01.

As mentioned above, we observed SAM depletion and downregulation of ATRAP expression in the tissues of NAFLD patients. Therefore, we suspected that SAM may positively regulate ATRAP levels, which means that exogenous SAM supplementation may ameliorate this condition. To verify our speculation, we induced NAFLD in 21 male C57BL/6 rats, which were divided into 3 groups (7 in each group), namely, the control group (balanced diet), HF group (high-fat diet), and HF plus SAM group (high-fat diet with SAM supplementation)^27^. After sacrifice, the liver tissues of all groups were harvested and analyzed. By observing histological images of liver tissues collected from the experimental rats, we noticed significant lipid droplet accumulation in the liver tissues of rats from the HF group. As expected, the rats that received exogenous SAM supplementation exhibited less steatosis (Fig. 2A). Next, we assessed the SAM concentration in liver tissues via UPLC. The achieved outcomes indicated that SAM was depleted in the HF group and upregulated upon exogenous supplementation (Fig. 2B). We then measured the ATRAP protein level by western blotting and immunohistochemistry. ATRAP expression was downregulated in the HF group and could be restored by SAM treatment (Fig. 2C, D). Thus, we demonstrated that SAM, which was depleted in NAFLD, could positively maintain ATRAP protein levels in vivo.

### SAM controlled ATRAP mRNA nucleocytoplasmic shuttling in the liver

**Fig. 3:**
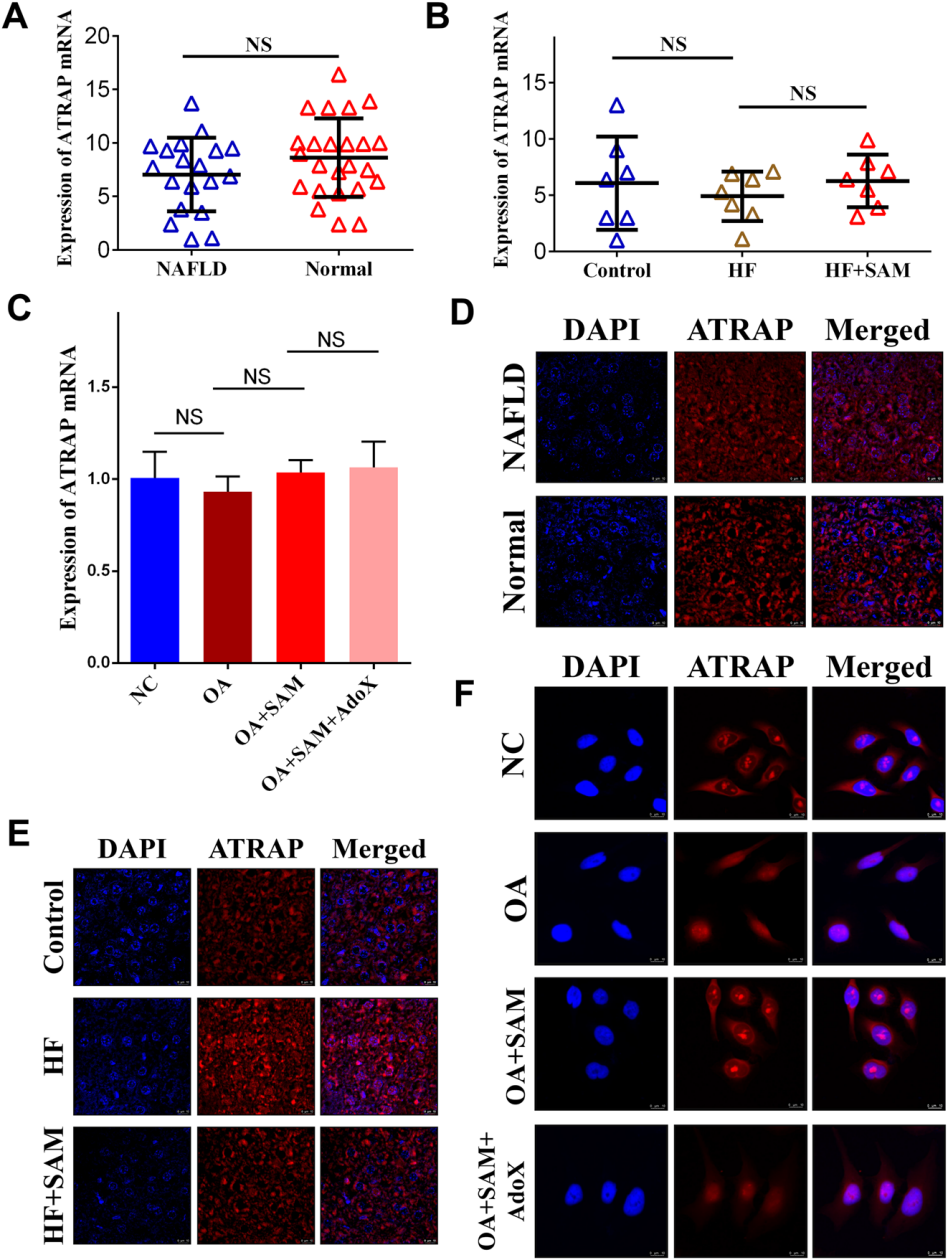
ATRAP mRNA translocation in NAFLD. **A** ATRAP mRNA levels in NAFLD and normal liver tissues, as determined by q-PCR. **B** ATRAP mRNA levels in the liver tissues of rats in the control, HF, and HF plus SAM groups. **C** The expression of ATRAP mRNA in L02 cells after OA, OA plus SAM, and OA plus SAM plus AdoX treatment. The error bars are the SDs (*n* = 3). **D** Fluorescence in situ hybridization images showing ATRAP mRNA in NAFLD and normal liver tissues (bar = 10 μm). **E** RNA FISH detection of ATRAP mRNA in the livers of rats from the control, HF, and HF plus SAM groups (bar = 10 μm). **F** FISH images showing the subcellular localization of ATRAP mRNA in L02 cells treated with OA, OA plus SAM, and OA plus SAM plus AdoX (bar = 10 μm). NS not significant.

We illustrated that SAM restores ATRAP protein expression in vivo, but the molecular mechanisms underlying the process by which SAM upregulates ATRAP are still not known. To investigate these mechanisms, we analyzed ATRAP mRNA levels in clinical samples by qRT-PCR. Unexpectedly, the results indicated that ATRAP mRNA levels in NAFLD tissues were similar to those in normal liver tissues (Fig. 3A). We next evaluated relative ATRAP mRNA levels in rat liver tissues by qPCR. Similarly, there was no difference between the control and HF groups. Moreover, no considerable difference was detected between the HF and HF plus SAM groups (Fig. 3B). To further verify the above phenomenon in vitro, we constructed an NAFLD model in vitro by treating the normal human hepatic L02 cells with OA^28^ (Supplementary Fig. S1). Then we observed the ATRAP mRNA expression after treating exogenous SAM and methylation inhibitor AdoX by qPCR. The outcomes illustrated that the levels of ATRAP mRNA were not altered after OA or OA plus SAM or OA plus SAM plus AdoX treatment (Fig. 3C), although we demonstrated that SAM could enhance ATRAP protein levels. Therefore, we deduced that posttranscriptional regulation, such as mRNA nucleocytoplasmic shuttling, may underlie this process. To verify this speculation, we visualized ATRAP mRNA and its subcellular localization in the clinical samples by FISH. The results revealed that ATRAP mRNA was trapped in the nucleus in tissues from patients with NAFLD (Fig. 3D). Next, we performed FISH to detect the subcellular localization of ATRAP mRNA in the rat liver samples. As expected, we found that ATRAP mRNA was trapped in the nucleus in the HF group and that this nuclear localization of ATRAP mRNA could be reversed by SAM supplementation (Fig. 3E). Finally, by utilizing FISH, we observed that ATRAP mRNA was trapped in the nucleus in L02 cells treated with OA. More importantly, SAM could ameliorate this abnormal subcellular localization, which could be antagonized by AdoX (Fig. 3F). Taken together, these results suggest that NAFLD-induced SAM depletion caused abnormal subcellular localization of ATRAP mRNA, which indicated that SAM could upregulate ATRAP protein expression by maintaining the nucleocytoplasmic shuttling of ATRAP mRNA.

### HuR played an important role in ATRAP mRNA nucleocytoplasmic shuttling

**Fig. 4:**
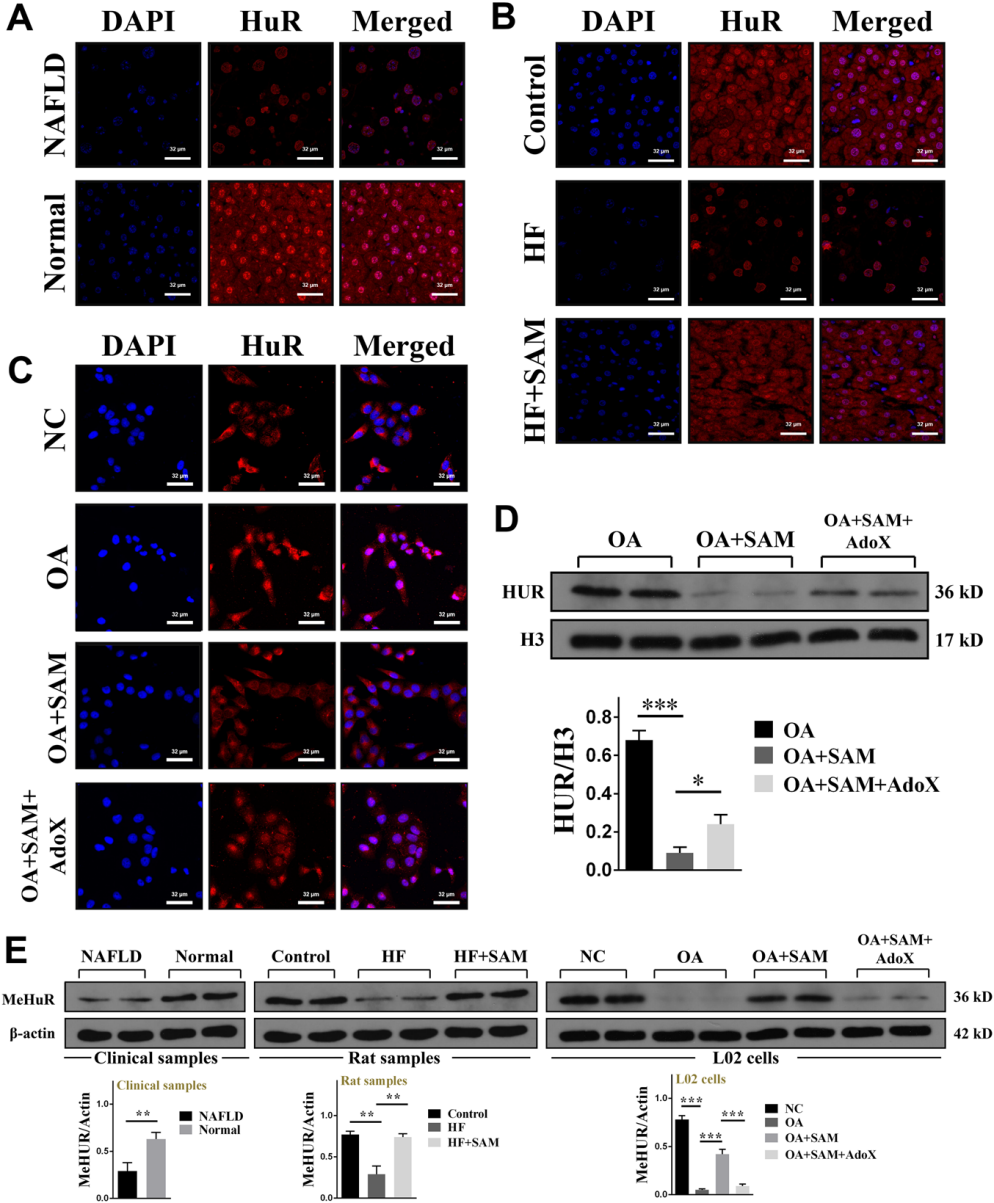
The methylation status and subcellular localization of the HuR protein were altered in NAFLD. **A** Detection of the HuR protein in NAFLD and normal liver tissues by immunofluorescence staining (bar = 32 μm). **B** Detection of the HuR protein in rats from the control, HF, and HF plus SAM groups by immunofluorescence (bar = 32 μm). **C** Subcellular localization of the HuR protein in L02 cells treated with OA, OA plus SAM, and OA plus SAM plus AdoX, as determined by immunofluorescence (bar = 32 μm). **D** Nuclear levels of the HuR protein in L02 cells after treatment with OA, OA plus SAM, or OA plus SAM plus AdoX, as determined by western blotting. The error bars are the SDs (*n* = 3). **E** Methylated HuR protein in normal, NAFLD, or SAM-treated liver tissues or cells in vivo and in vitro, as determined by western blotting. The error bars are the SDs (*n* = 3). *P < 0.05, **P < 0.01, ***P < 0.001.

We confirmed that SAM regulates the subcellular localization of ATRAP mRNA to affect its protein level, but the specific mechanism of ATRAP mRNA shuttling is still unknown. We speculated that mRNA shuttling is regulated by certain RNA-binding proteins (RBPs), which may also be controlled by SAM. To confirm our hypothesis, we first predicted the RBP that targets ATRAP mRNA by using the online starBase 2.0 and starBase 3.0 tool^29^. As predicted, we observed that HuR might interact with ATRAP mRNA by directly binding to it, which indicates that HuR may be regulated by SAM (Supplementary Tables S3 and S4). To prove this hypothesis, we first conducted immunofluorescence to observe the subcellular localization of HuR in the clinical samples. We discovered that HuR was placed in both the nucleus and cytoplasm in normal liver tissues but was trapped in the nucleus in the tissues of patients with NAFLD (Fig. 4A). After that, we further performed immunofluorescence for the HuR protein in model rats. The results showed that HuR was mainly located in the nucleus in the livers of rats fed a high-fat diet. Moreover, this aberrant localization could be corrected by SAM supplementation (Fig. 4B). These in vivo results were verified in vitro by utilizing the abovementioned L02 cells. The cells were cultured with OA or OA plus SAM or OA plus SAM plus AdoX, and we found that HuR was abnormally located in the nucleus in cells treated with OA and that this abnormal localization could be reversed by SAM supplementation. Similarly, the effects of SAM could be offset by AdoX (Fig. 4C). We next semiquantitatively analyzed HuR protein levels in the nucleus through western blotting. The outcomes indicated that, under OA treatment, the amount of intranuclear HuR was significantly higher than that after SAM treatment. But the HuR protein in the nucleus was accumulated again after AdoX treatment (Fig. 4D). Thus, we demonstrated that the SAM concentration could directly influence the localization of HuR and that this effect was likely due to posttranslational protein modification. Meanwhile, this modification could be antagonized by AdoX. We know that chemical modification of proteins can alter their function and activity, including inducing changes in subcellular localization^30^. SAM is an active methyl donor; therefore, we hypothesized that the SAM concentration may alter HuR methylation to affect its subcellular localization. To confirm our speculation, we performed western blotting to analyze the degree of methylation of the HuR protein using a previously prepared anti-methyl HuR antibody^31^. The results determined that HuR was demethylated in the in vivo and in vitro models (Fig. 4E). Therefore, we concluded that HuR was regulated by SAM and exhibited the same subcellular localization as ATRAP mRNA. Thus, we concluded that HuR played an important role and might participate in ATRAP mRNA nucleocytoplasmic shuttling.

### SAM maintained ATRAP mRNA nucleocytoplasmic shuttling by maintaining HuR methylation

**Fig. 5:**
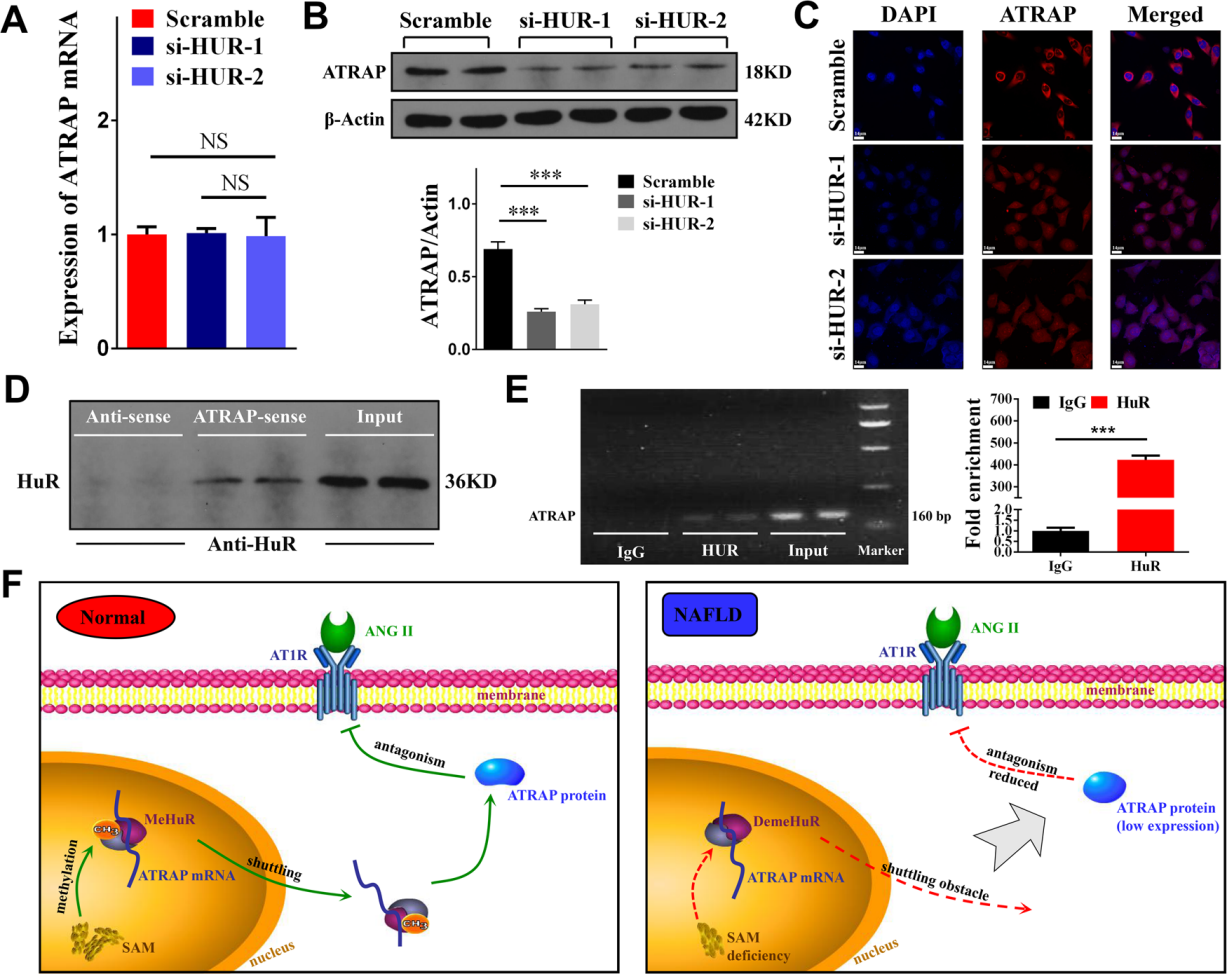
HuR could directly bind ATRAP mRNA and regulate its nucleocytoplasmic shuttling. **A** Measurement of ATRAP mRNA levels by RT-PCR after transfection of L02 cells with HuR-siRNA. The error bars are the SDs (*n* = 3). **B** The ATRAP protein level after HuR knockdown, as determined by western blotting. The error bars are the SDs (*n* = 3). **C** FISH images showing ATRAP mRNA after HuR knockdown in L02 cells (bar = 14 μm). **D** Western blot analysis of the specific association of ATRAP mRNA with HuR in RNA pull-down assays. **E** RNA immunoprecipitation (RIP) experiments were performed using an HuR antibody, and specific primers were used to detect ATRAP mRNA by qPCR. The error bars are the SDs (*n* = 3). **F** Schematic of the proposed mechanism by which SAM upregulates ATRAP expression via HuR methylation to maintain ATRAP mRNA nucleocytoplasmic shuttling. NS not significant, ***P < 0.001.

The subcellular localization of HuR was proven to be determined by the SAM concentration. However, the direct interaction between HuR and ATRAP mRNA was not addressed. According to our hypothesis, a low SAM concentration resulted in demethylation of HuR to hinder ATRAP mRNA nucleocytoplasmic shuttling. Therefore, we performed qPCR and western blotting to measure ATRAP expression after knocking down HuR in L02 cells. The results indicated that HuR could positively regulate ATRAP protein expression but had no influence on the mRNA level of ATRAP (Fig. 5A, B). We next conducted RNA FISH to visualize ATRAP mRNA localization after knocking down HuR. We observed a decrease in ATRAP mRNA cytoplasmic localization (Fig. 5C). Finally, we performed RNA pull down and RIP-PCR to confirm the direct binding between ATRAP mRNA and HuR. The results proved that ATRAP mRNA interacted with HuR by direct binding (Fig. 5D, E). Taken together, these results demonstrated that SAM could upregulate ATRAP protein expression. Regarding the molecular mechanisms, ATRAP mRNA nucleocytoplasmic shuttling relied on HuR methylation, which was determined by the SAM concentration in the liver (Fig. 5F).

## Discussion

We first investigated the SAM concentration and ATRAP protein levels in clinical liver samples and discovered that the expression of both SAM and ATRAP as downregulated in NAFLD samples, as in animal models. Then we demonstrated that SAM supplementation reduced steatosis and that SAM had positive effects on upregulating ATRAP protein expression. We unexpectedly found that SAM could enhance ATRAP protein expression without influencing its mRNA level. Thus, we considered mRNA nucleocytoplasmic shuttling a potential mechanism and confirmed this mechanism in vivo and in vitro. After that, we predicted that HuR might be involved in the regulatory effects of SAM on ATRAP mRNA. Finally, HuR protein methylation and subcellular localization were also proven to be determined by the SAM concentration, and ATRAP mRNA shuttling was shown to rely on the HuR protein by direct binding. Therefore, we concluded that SAM positively regulates ATRAP in NAFLD and that HuR is involved in this process.

SAM is a principal biological methyl donor that participates in many intracellular biochemical processes^32^. It has been determined to be effective for chronic liver diseases and HCC^22,33^. In animal experiments, SAM has repeatedly been determined to be involved in NAFLD, but determining the molecular mechanism is complicated because SAM participates in many metabolic circulation pathways^34^. However, the clinical benefit of SAM remains controversial, as there have been few studies and few clinical trials^24^. In contrast, ATRAP has been well investigated in the kidney, heart, and testis, and high expression is observed in these tissues^15^. With the deepening of basic research, the role of RAS in NAFLD was discovered in recent years, but the role of ATRAP in NAFLD has been rarely reported due to its lower expression in the liver than in the abovementioned tissues^10,15^. As a novel molecule that is able to inhibit AT1R signaling to suppress Ang II-induced hypertrophic and proliferative responses, ATRAP and its effects have been recently considered^17,18^.

In the current research, we found that SAM expression was downregulated in NAFLD, which was consistent with previous results^26,35,36^. SAM is known to be enriched in the liver, and up to 85% of all the reactions of transmethylation take place in the liver^37^. Patients with liver injury, such as fibrosis and hepatitis, have decreased expression of MAT1A and hepatic SAM levels, which contributes to diminished hepatic glutathione (GSH) levels in these cases. Administration of SAM has been shown to normalize GSH levels in cases with either nonalcoholic or alcoholic liver disease^38,39^. These are important theoretical bases for the use of SAM as a treatment for NAFLD^40^. Moreover, scientists have realized that MAT1A is underexpressed in cases with advanced NAFLD, obviously insisting on a leading role for SAM in the pathogenesis of NAFLD in humans^41,42^. However, the initial evidence of the advantageous effects of SAM in the models of NASH originated from animals fed a methionine–choline-deficient diet^25^. Therefore, the role of SAM likely involves sensitization of the liver to release pro-inflammatory cytokines due to a reduced hepatic SAM level, which may be prevented by SAM treatment^43^. Thus, some studies have suggested that SAM may have benefits only if the SAM level is decreased or in the later stages of NAFLD^24^. However, in our research, the patients and rats indeed exhibited a reduction in SAM levels, and NAFLD was ameliorated by SAM supplementation. Thus, we speculated that SAM may exert more effects than previously thought; however, we admit that the specific efficacy of SAM in NAFLD requires further investigation. Nevertheless, we confirmed that the effects of SAM in the current research were associated with its regulation of ATRAP. It was previously determined that ATRAP antagonizes AT1R to inhibit fibrosis induced by Ang II^15,18^, which led to a focus on the late stage of NAFLD. However, Ang II participates in many processes in NAFLD, including lipid metabolism and insulin resistance, and NAFLD progression is continuous and involves different biological reactions^44–48^. Thus, we speculate that ATRAP, as an Ang II blocker, performs an essential task in the whole process of NAFLD. This is a new basis of the use of SAM for NAFLD treatment.

Notably, we observed that the RBP HuR takes part in the regulation of ATRAP by SAM. HuR is well established for attaching to a subset of mRNAs and for affecting their translation and/or stability; however, it has also been involved in nuclear export of mRNAs and target pre-mRNA splicing^49,50^. HuR plays multiple roles in different molecular events. At the same time, the subcellular distribution/modification of HuR, which may determine its functions, was also diversified in different tissues or cell lines^51–53^. Nevertheless, an important factor affecting the subcellular localization of HuR is its protein modification, such as phosphorylation and methylation^31,54^. For methylation, the enzymes that catalyze protein methylation, well known as protein arginine methyltransferases, are able to transfer methyl groups from SAM to a target protein, producing monomethylated and dimethylated amino acids^55^. Methylation of protein can regulate various cellular procedures, including transcription, protein–protein interactions, splicing, nuclear–cytoplasmic transport, and signaling^56–59^. In our study, we noticed that HuR was distributed in both the cytoplasm and nucleus in normal liver tissue and was trapped in nucleus after SAM deduction. Normally, the methylation of HuR protein occurs at Arg^217^ ^52^ and SAM, which presenting high concentration in the normal liver tissue, is synthesized in the liver^19,32^. These may be important conditions for HuR to shuttle between nucleus and cytoplasm in the dynamic equilibrium in hepatocytes. In other words, low SAM concentration induced by NAFLD leads to the occurrence of HuR demethylation, which directly breaks the dynamic balance of nucleocytoplasmic shuttling. Thus, HuR was trapped in the nucleus and affected the downstream nuclear shuttling of ATRAP mRNA. This indicated that HuR plays an important role in maintaining mRNA shuttling and is indispensable for NAFLD. The role of HuR may be a new future research direction in this field.

As mentioned above, the efficacy of SAM for the treatment of NAFLD has been reported, but the understanding of the associated molecular mechanisms is largely insufficient. The regulatory effects of SAM on ATRAP were addressed in this research for the first time. The current research provides a good theoretical basis for the treatment of NAFLD with SAM. According to existing theories and our own experimental findings, it may have a good therapeutic effect. Moreover, we found that the mechanism of SAM depletion mediates the alleviation of NAFLD by ATRAP. More importantly, this study demonstrated that, in normal liver tissues, a high concentration of SAM is important for the maintenance of RNA transport by HuR. However, we must admit that there are some inevitable drawbacks to the current research. First, the included clinical tissues and animal models may not perfectly represent the population due to the limited sample size. However, our results indeed demonstrate that SAM is beneficial for subjects with NAFLD with lower SAM levels. Moreover, since we enrolled surgical patients, most of them did not show a severe abnormal preoperative liver function, which may lead to a poor representation of NAFLD, especially NASH. Therefore, whether the association of SAM and ATRAP has great impacts in different RAS-mediated stages of NAFLD was not addressed. ATRAP is mainly targeted at AT1R, but other Ang II receptors were not examined in this study. Other downstream reactions involving other receptors or systems still need to be investigated in the future.

In summary, although there are some limitations to the current study, we provide basic medical evidence to support the hypothesis that SAM positively regulates ATRAP in NAFLD by maintaining its mRNA nucleocytoplasmic shuttling. In addition, we uncovered the molecular mechanism and demonstrated that HuR is involved and plays an important role in this regulation. Notably, to some extent, downregulation of ATRAP expression is induced by SAM depletion, and ATRAP is involved in the RAS system, which implies that SAM supplementation in NAFLD may exert more effects than we previously thought.

## Supplementary information

Supplementary material

Figure S1
