# Supplementary material for: S-adenosylmethionine upregulates the angiotensin receptor-binding protein ATRAP via the methylation of HuR in NAFLD
Source: Cell Death Dis. 2021 Mar 22;12(4):306. doi: 10.1038/s41419-021-03591-1 (PMC7985363; doi:10.1038/s41419-021-03591-1)
Supplement: Supplementary file 1 — Supplementary material [file 41419_2021_3591_MOESM1_ESM.doc]

**Supplementary Tables**

Supplementary Table S1. Primer sequences and target sequences used in this research.

| **Gene** | **Sequence (5'-3')** |
| --- | --- |
| ATRAP-F | CCTGGGGCTGCATTGTATTC |
| ATRAP-R | CTGATGTGCACGATGTCCAG |
| β-actin-F | AGCGAGCATCCCCCAAAGTT |
| β-actin-R | GGGCACGAAGGCTCATCATT |
| HuR-siRNA-1 | GAACGAAUUUGAUCGUCAATT |
|  | UUGACGAUCAAAUUCGUUCTT |
| HuR-siRNA-2 | GCAGAUGUUUGGGCCGUUUTT |
|  | AAACGGCCCAAACAUCUGCTT |
| HuR-siRNA-control  (scramble) | UUCUCCGAACGUGUCACGUTT |
|  | ACGUGACACGUUCGGAGAATT |

Supplementary Table S2. Antibodies used in this study.

| **Antibody** | **Company** | **Cat No.** | **Western Blot** | **IHC** | **IF** |
| --- | --- | --- | --- | --- | --- |
| β-actin | Proteintech | 60008-1-Ig | 1:2000 |  |  |
| ATRAP | Santa Cruz | sc-271367 | 1:2000 |  |  |
| ATRAP | Proteintech | 11559-1-AP |  | 1:100 |  |
| HuR | Proteintech | 11910-1-AP | 1:2000 |  | 1:200 |
| H3 | Abcam | ab-1791 | 1:1000 |  |  |

**Supplementary Table S3.** Predicted RBPs of ATRAP mRNA in Starbase 2.0 (http://starbase.sysu.edu.cn/starbase2/index.php)

| **RBP** | **Gene Name** | **Target Sites** | **Bio Complex** | **Clip Read Num** | **Cancer Num** |
| --- | --- | --- | --- | --- | --- |
| HuR | AGTRAP | 8 | 1 | 8 | 11 |
| PTB | AGTRAP | 6 | 1 | 137 | 10 |
| IGF2BP1 | AGTRAP | 3 | 1 | 26 | 5 |
| IGF2BP2 | AGTRAP | 1 | 1 | 5 | 9 |
| IGF2BP3 | AGTRAP | 2 | 1 | 22 | 8 |
| eIF4AIII | AGTRAP | 21 | 2 | 107 | 10 |
| DGCR8 | AGTRAP | 2 | 1 | 7 | 9 |
| FMRP | AGTRAP | 8 | 3 | 8000 | 12 |
| LIN28A | AGTRAP | 1 | 1 | 11 | 3 |
| CAPRIN1 | AGTRAP | 1 | 1 | 519 | 9 |
| U2AF65 | AGTRAP | 2 | 1 | 8 | 9 |
| UPF1 | AGTRAP | 38 | 3 | 232 | 11 |

**Supplementary Table S4.** Predicted RBPs of ATRAP mRNA in Starbase 3.0 (http://starbase.sysu.edu.cn/).

| **RBP** | **Gene Name** | **Gene Type** | **Cluster Num** | **Clip Exp Num** | **Pancancer Num** |
| --- | --- | --- | --- | --- | --- |
| ADAR | AGTRAP | protein_coding | 1 | 1 | 15 |
| AIFM1 | AGTRAP | protein_coding | 2 | 1 | 18 |
| AUH | AGTRAP | protein_coding | 1 | 1 | 15 |
| BCCIP | AGTRAP | protein_coding | 2 | 2 | 16 |
| BUD13 | AGTRAP | protein_coding | 1 | 1 | 17 |
| CAPRIN1 | AGTRAP | protein_coding | 1 | 1 | 25 |
| CELF2 | AGTRAP | protein_coding | 1 | 1 | 18 |
| CNBP | AGTRAP | protein_coding | 1 | 1 | 17 |
| CPSF6 | AGTRAP | protein_coding | 3 | 2 | 27 |
| CSTF2T | AGTRAP | protein_coding | 2 | 2 | 20 |
| DDX3X | AGTRAP | protein_coding | 4 | 2 | 28 |
| DDX42 | AGTRAP | protein_coding | 1 | 1 | 27 |
| DDX54 | AGTRAP | protein_coding | 1 | 1 | 20 |
| DGCR8 | AGTRAP | protein_coding | 5 | 3 | 19 |
| DHX9 | AGTRAP | protein_coding | 2 | 1 | 25 |
| DKC1 | AGTRAP | protein_coding | 2 | 1 | 16 |
| EIF4A1 | AGTRAP | protein_coding | 1 | 1 | 20 |
| EIF4A3 | AGTRAP | protein_coding | 38 | 2 | 15 |
| EIF4G1 | AGTRAP | protein_coding | 1 | 1 | 12 |
| EIF4G2 | AGTRAP | protein_coding | 2 | 1 | 24 |
| ELAVL1 | AGTRAP | protein_coding | 12 | 2 | 16 |
| FAM120A | AGTRAP | protein_coding | 6 | 4 | 22 |
| FBL | AGTRAP | protein_coding | 14 | 2 | 21 |
| FMR1 | AGTRAP | protein_coding | 9 | 5 | 25 |
| FTO | AGTRAP | protein_coding | 1 | 1 | 24 |
| FUS | AGTRAP | protein_coding | 8 | 3 | 20 |
| FXR2 | AGTRAP | protein_coding | 3 | 1 | 15 |
| GNL3 | AGTRAP | protein_coding | 2 | 2 | 22 |
| GTF2F1 | AGTRAP | protein_coding | 3 | 3 | 17 |
| HNRNPA1 | AGTRAP | protein_coding | 4 | 4 | 23 |
| HNRNPA2B1 | AGTRAP | protein_coding | 2 | 2 | 20 |
| HNRNPC | AGTRAP | protein_coding | 8 | 6 | 19 |
| HNRNPK | AGTRAP | protein_coding | 9 | 5 | 20 |
| HNRNPL | AGTRAP | protein_coding | 1 | 2 | 20 |
| HNRNPM | AGTRAP | protein_coding | 4 | 4 | 16 |
| HNRNPUL1 | AGTRAP | protein_coding | 4 | 1 | 18 |
| IGF2BP1 | AGTRAP | protein_coding | 12 | 5 | 17 |
| IGF2BP2 | AGTRAP | protein_coding | 17 | 6 | 20 |
| IGF2BP3 | AGTRAP | protein_coding | 10 | 5 | 19 |
| KHDRBS1 | AGTRAP | protein_coding | 1 | 1 | 24 |
| KHSRP | AGTRAP | protein_coding | 1 | 1 | 13 |
| LARP4B | AGTRAP | protein_coding | 6 | 1 | 17 |
| LARP7 | AGTRAP | protein_coding | 2 | 2 | 23 |
| LIN28 | AGTRAP | protein_coding | 3 | 2 | 12 |
| LIN28A | AGTRAP | protein_coding | 1 | 1 | 12 |
| LIN28B | AGTRAP | protein_coding | 8 | 4 | 14 |
| LSM11 | AGTRAP | protein_coding | 1 | 1 | 26 |
| MOV10 | AGTRAP | protein_coding | 3 | 3 | 25 |
| MSI1 | AGTRAP | protein_coding | 1 | 1 | 14 |
| MSI2 | AGTRAP | protein_coding | 2 | 2 | 27 |
| NOP56 | AGTRAP | protein_coding | 8 | 1 | 16 |
| NOP58 | AGTRAP | protein_coding | 8 | 2 | 17 |
| NPM1 | AGTRAP | protein_coding | 2 | 1 | 19 |
| NUMA1 | AGTRAP | protein_coding | 3 | 2 | 18 |
| PCBP2 | AGTRAP | protein_coding | 4 | 2 | 17 |
| PRPF8 | AGTRAP | protein_coding | 5 | 4 | 21 |
| PTBP1 | AGTRAP | protein_coding | 14 | 4 | 19 |
| QKI | AGTRAP | protein_coding | 2 | 2 | 22 |
| RANGAP1 | AGTRAP | protein_coding | 2 | 2 | 29 |
| RBFOX2 | AGTRAP | protein_coding | 21 | 7 | 24 |
| RBM10 | AGTRAP | protein_coding | 4 | 2 | 19 |
| RBM22 | AGTRAP | protein_coding | 3 | 2 | 16 |
| RBM27 | AGTRAP | protein_coding | 2 | 1 | 26 |
| RBM5 | AGTRAP | protein_coding | 1 | 1 | 21 |
| RNF219 | AGTRAP | protein_coding | 2 | 1 | 26 |
| SAFB2 | AGTRAP | protein_coding | 3 | 2 | 20 |
| SBDS | AGTRAP | protein_coding | 1 | 1 | 21 |
| SF3A3 | AGTRAP | protein_coding | 5 | 2 | 20 |
| SF3B4 | AGTRAP | protein_coding | 4 | 2 | 17 |
| SLBP | AGTRAP | protein_coding | 1 | 1 | 16 |
| SLTM | AGTRAP | protein_coding | 2 | 2 | 26 |
| SMNDC1 | AGTRAP | protein_coding | 3 | 3 | 22 |
| SND1 | AGTRAP | protein_coding | 4 | 3 | 16 |
| SRSF1 | AGTRAP | protein_coding | 7 | 5 | 23 |
| SRSF10 | AGTRAP | protein_coding | 2 | 1 | 20 |
| SRSF3 | AGTRAP | protein_coding | 3 | 1 | 17 |
| SRSF7 | AGTRAP | protein_coding | 4 | 1 | 20 |
| SRSF9 | AGTRAP | protein_coding | 3 | 2 | 18 |
| TAF15 | AGTRAP | protein_coding | 2 | 2 | 17 |
| TARDBP | AGTRAP | protein_coding | 7 | 4 | 20 |
| TIA1 | AGTRAP | protein_coding | 2 | 2 | 26 |
| TNRC6A | AGTRAP | protein_coding | 2 | 2 | 28 |
| TRA2A | AGTRAP | protein_coding | 1 | 2 | 19 |
| TROVE2 | AGTRAP | protein_coding | 3 | 1 | 25 |
| U2AF1 | AGTRAP | protein_coding | 3 | 3 | 17 |
| U2AF2 | AGTRAP | protein_coding | 18 | 7 | 19 |
| UPF1 | AGTRAP | protein_coding | 24 | 4 | 16 |
| XRN2 | AGTRAP | protein_coding | 2 | 2 | 20 |
| YTHDC1 | AGTRAP | protein_coding | 2 | 3 | 26 |
| YTHDF1 | AGTRAP | protein_coding | 1 | 1 | 17 |
| ZNF184 | AGTRAP | protein_coding | 1 | 2 | 25 |

**Supplementary Figure legend**

**Figure S1.** The NAFLD model was determined by FISH with BODIPY® lipid probes in L02 cells in vitro induced by OA (bar=25 μm).
