# Supplementary figures and images for: S-adenosylmethionine upregulates the angiotensin receptor-binding protein ATRAP via the methylation of HuR in NAFLD
Source: Cell Death Dis. 2021 Mar 22;12(4):306. doi: 10.1038/s41419-021-03591-1 (PMC7985363; doi:10.1038/s41419-021-03591-1)

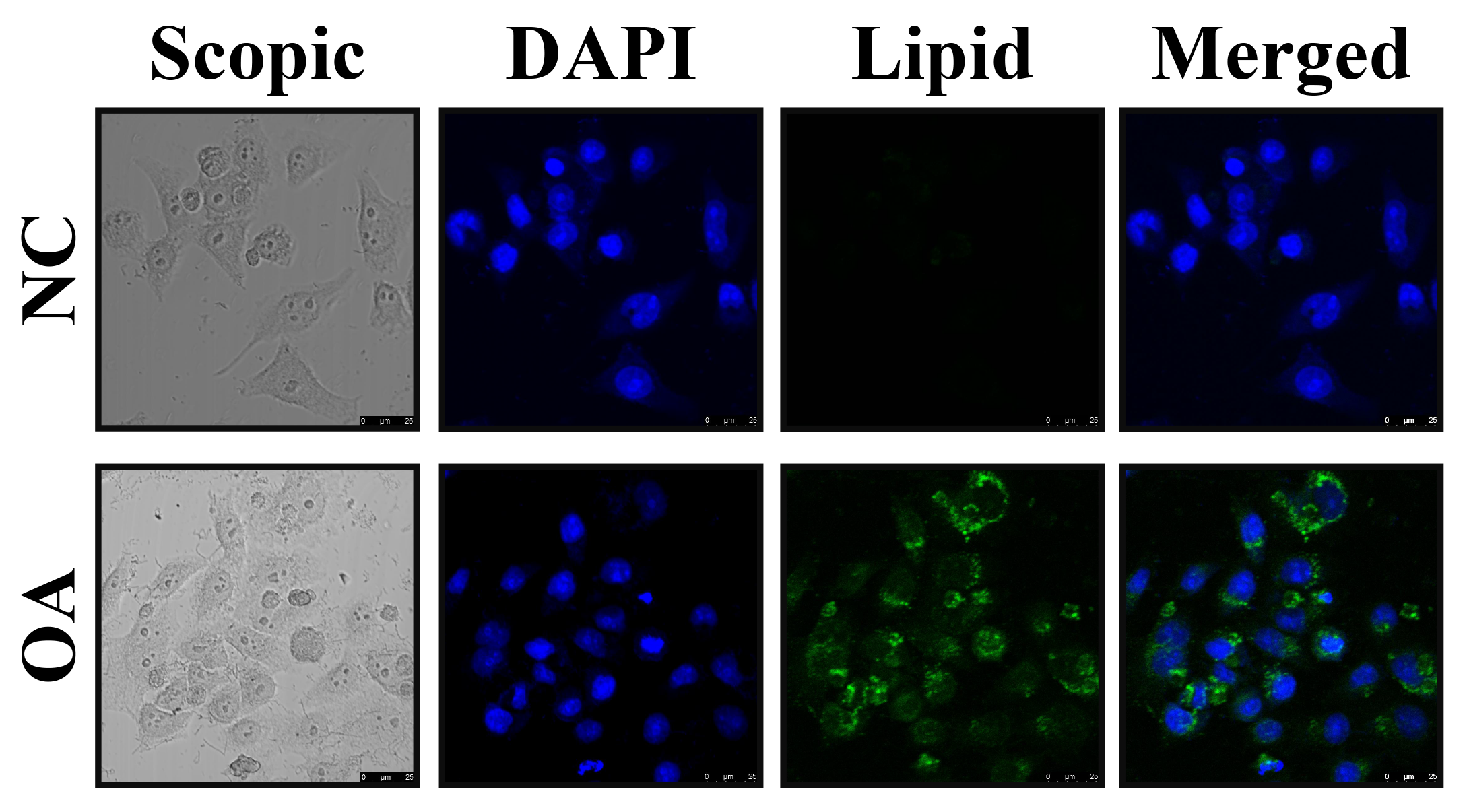

Supplement: Supplementary file 2 — Figure S1 [file 41419_2021_3591_MOESM2_ESM.tif]
